# Biotic Resistance to an Alien Amphibian: Larval Competition between Japanese Frogs and Invasive Cane Toads

**DOI:** 10.1371/journal.pone.0156396

**Published:** 2016-06-02

**Authors:** Takashi Haramura, Hirohiko Takeuchi, Michael R. Crossland, Richard Shine

**Affiliations:** 1 The Hakubi Center for Advanced Research, Kyoto University, Sakyo, Kyoto, 606–8501, Japan; 2 Field Science Education and Research Center, Kyoto University, Shirahama, Wakayama, 649–2211, Japan; 3 School of Life and Environmental Sciences A08, University of Sydney, Sydney, NSW 2006, Australia; State Natural History Museum, GERMANY

## Abstract

Understanding negative effects of native species on introduced taxa may suggest novel ways to control the invasive species by enhancing such effects. Previous studies have reported that the larvae of invasive cane toads (*Rhinella marina*) are suppressed by competition with the larvae of native anurans in Australia, but not in North America. We conducted laboratory trials to measure the effect of exposure to the larvae of Japanese frogs (*Microhyla ornata*, *Fejervarya sakishimensis*, *Rhacophorus owstoni*) on rates of survival, growth and development of cane toad tadpoles in Ishigaki Island, in southern Japan. Survival rates were not affected by native species, but competition with Dicroglossids and Rhacophorids (but not Microhylids) strongly reduced rates of growth and development in the tadpoles of cane toads. Dicroglossid tadpoles also reduced the body condition to toad tadpoles in addition to effects on SVL and mass. Encouraging populations of native frogs in toad-invaded areas of Japan thus may help to reduce the numbers of invasive cane toads.

## Introduction

The introduction of species to areas outside their native range creates a wide range of novel biological interactions, because the invader encounters a new suite of potential prey items, predators and competitors. In some cases, those interactions may inhibit the successful establishment and expansion of invader populations; and we may be able to exploit such biotic resistance to fashion effective new approaches to biocontrol [1–5]. Importantly, using native taxa for this purpose avoids many of the risks associated with implementing biocontrol based upon introducing additional invaders, or directly manipulating other aspects of the ecosystem [6]. Before we can frame such strategies, however, we need to know whether or not the invader’s numbers are indeed negatively affected by interactions with native fauna.

Both intraspecific and interspecific competition are intense among anuran larvae in small waterbodies [7], leading to the evolution of diverse mechanisms for suppression of competing larvae [8,9]. Plausibly, then, the tadpoles of an invasive anuran might experience lower viability if they develop sympatrically with the larvae of native anurans. Studies on invasive cane toads (*Rhinella [Bufo] marina*) in Australia have reported that the larvae of native anurans can suppress survival and/or growth of toad larvae both in the laboratory and in the field [7, 10–13], whereas similar studies in North America reported no such effects [14]. We conducted experiments to determine whether or not the tadpoles of invasive cane toads are negatively affected by the presence of native anuran larvae in another area to which this large amphibian has been introduced: Ishigaki Island in southern Japan.

## Materials and Methods

### Ethics Statement

Because eggs and tadpoles used in this experiment were not collected in a protected area, no permit was required from the relevant wildlife regulatory agency. The owners of the paddy fields from which tadpoles were collected gave permission for us to collect those specimens. Collection of eggs and tadpoles was done using small mesh nets, in accordance with the field research guideline of the Japan Ethological Society. The number of animals used was restricted to the minimum needed to achieve statistically robust comparisons. The study animal (*Rhinella marina*) is designated as a pest species; the Ministry of the Environment of Japan has issued a permit to us to study them. None of the other three species (*Microhyla ornata*, *Fejervarya sakishimensis*, *Rhacophorus owstoni*) are endangered or protected. All animal care procedures were authorised by the Ministry of the Environment of Japan (14000279), and experiments were conducted under the regulation of Kyoto University Ethics Committee.

### Methods

Ishigaki is one of the southernmost islands within an archipelago that stretches southwest from Okinawa (24˚36´52´´N, 124˚15´62´´E). Ishigaki Island experiences a subtropical climate, and anuran tadpoles occur in a wide range of waterbodies such as paddy fields, ponds, and artificial pools in sugarcane fields. Cane toads are large (to > 1 kg as adults) bufonid anurans, native to South and Central America, that have been introduced to many places worldwide to control insect pests in commercial agriculture [15]. Approximately 10 to 15 individuals were brought to Ishigaki Island in 1978 to control sugar cane pests [16].

Ishigaki Island contains about eight species of native anurans, most of which include aquatic eggs and larvae in their life cycles [17]. Our laboratory studies included tadpoles of three Families, as follows:

1. Microhylidae. The ornate narrow-mouthed frog (*Microhyla ornata*) is the smallest (adults 22–32 mm snout-urostyle length, SUL) of the local anurans, often found in leaf litter.
2. Dicroglossidae. The Sakishima rice frog (*Fejervarya sakishimensis*) is a larger ground-dwelling anuran (adults 41–70 mm SUL).
3. Rhacophoridae. The Owston’s green tree frog (*Rhacophorus owstoni*) is similar in size to the preceding species, but arboreal in habit.

Tadpoles of *Microhyla ornata* and *Rhacophorus owstoni* were derived from eggs collected in natural waterbodies, whereas tadpoles of *Fejervarya sakishimensis* were collected in paddy fields. These waterbodies are also used by cane toads as breeding sites (Haramura, personal observation). Cane toad tadpoles were obtained by injecting local field-collected adults with 0.25 mg mL^-1^ of leuprorelin acetate (Leuplin, Takeda Pharmaceutical Company, Japan), to induce amplexus and spawning. Tadpoles of all four species were maintained in groups in 120 L plastic containers (66 x 86 x 34 cm). Tadpoles were fed algal pellets (Hikari Algae Wafers, Kyorin) ad libitum, and water was changed weekly. The tadpoles used in the experiment were haphazardly selected from these containers and added to experimental bins as described below.

Experiments were conducted using plastic bins (38 x 55 x 30 cm), each filled with 60 L water and located in a covered building exposed to ambient temperatures. At the start of the experiment, we added a 2 cm layer of soil substrate and 3 g of algal pellets to each bin. Tadpoles varied in sizes and developmental stages at the beginning of the experiment (see Table 1).

**Table 1:** Body sizes (mean ± standard errors, and range) and Gosner stages for tadpoles as measured at the beginning of the experiments.

	Body size (mm)	Mass (g)	Gosner stage
*Rhinella [Bufo] marina*	3.81 ± 0.11 (3.17–4.26)	0.015 ± 0.008 (0.004–0.010)	24.1 ± 0.23 (23–25)
*Microhyla ornata*	4.32 ± 0.16 (3.60–5.17)	0.015 ± 0.001 (0.008–0.025)	24.0 ± 0.29 (23–25)
*Fejervarya sakishimensis*	5.24 ± 0.59 (3.35–8.74)	0.027 ± 0.008 (0.006–0.092)	24.4 ± 0.52 (23–28)
*Rhacophorus owstoni*	5.52 ± 0.21 (4.28–6.22)	0.017 ± 0.001 (0.006–0.025)	24.5 ± 0.34 (23–26)

Our experiments included six treatments (with six replicates per treatment): three different densities of *R*. *marina* tadpoles (10, 20 or 40 per container); and 10 *R*. *marina* plus 10 tadpoles of either *M*. *ornata*, *F*. *sakishimensis*, or *R*. *owstoni* per container. We did not include additional treatments with native tadpoles alone, because of limited availability and because our focus was on the impact of native tadpoles on cane toad tadpoles rather than *vice versa*. After 20 days, we terminated the experiment and recorded number of surviving tadpoles in each tank. For treatments containing 10 toad tadpoles, we measured the snout-vent length (SVL), mass and developmental stage [18] of all surviving toad tadpoles in the tank. Tadpole SVL and mass were generally highly correlated. However, to clarify potential treatment effects on tadpole body shape, we have retained information on both of these traits. For treatments containing 20 or 40 toad tadpoles, we measured these traits on 10 haphazardly chosen individuals.

Using JMP Pro 11.2 (SAS Institute 2013), we analysed growth and development data using one-factor ANOVA with treatment as the factor, and size and developmental stage as dependent variables (using mean values per container to avoid pseudoreplication). Survival data were analysed as a binomial response using logistic regression (logit link function) in R [19]. To examine treatment effects on body shape, we fit linear regression to all tadpole SVL and mass data to obtain residuals, and then analysed treatment effects on tank residual means using ANOVA.

Data from this study have been deposited in Dryad (DOI: http://dx.doi.org/10.5061/dryad.rn192)

## Results

**Fig 1:**
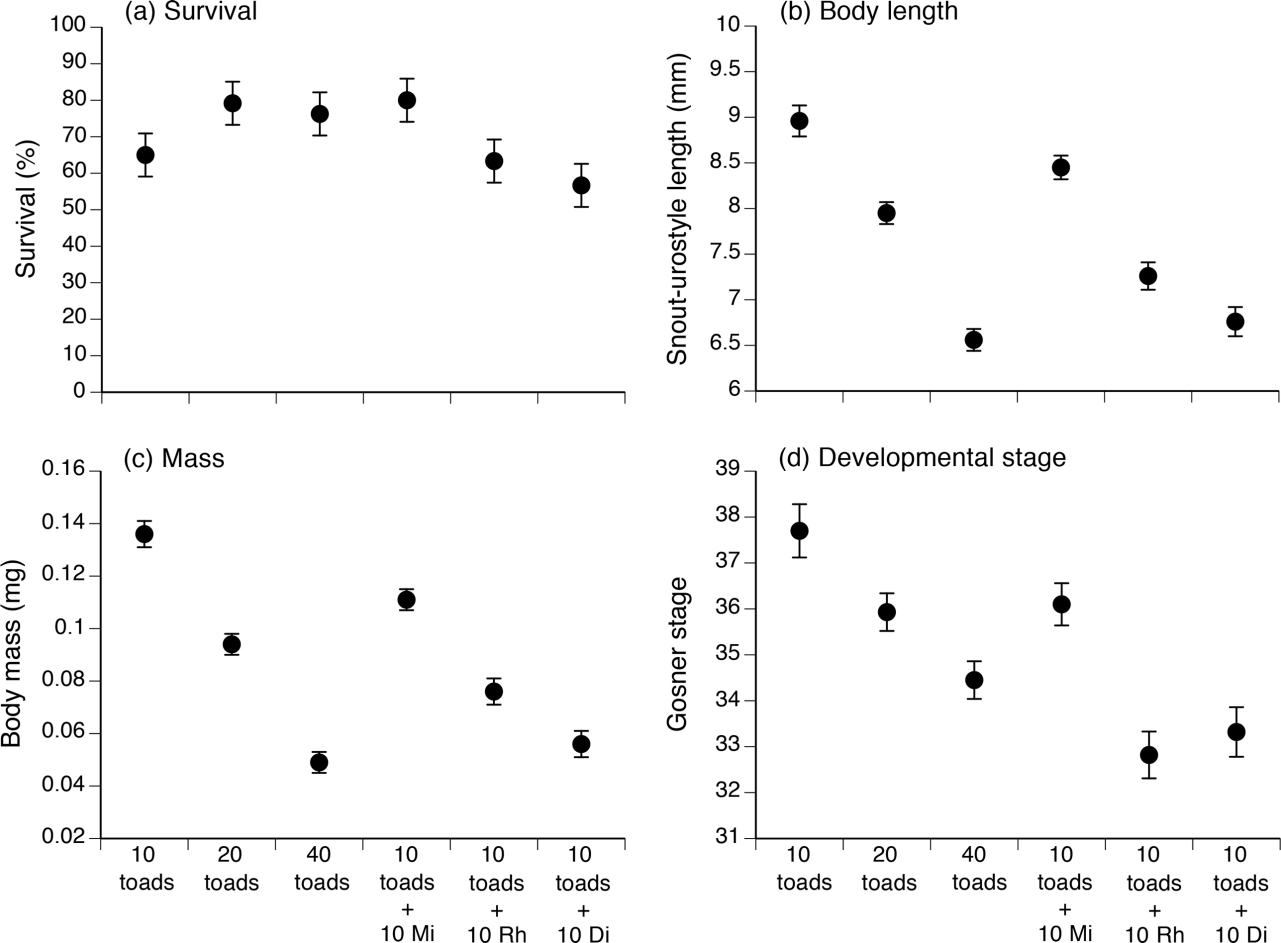
The effect of the presence of native frog tadpoles on survival, growth and development of tadpoles of the invasive cane toad, as measured 20 days after experimental exposure. The treatments comprised 10, 20 or 40 cane toad tadpoles, or 10 cane toad tadpoles plus 10 of either microhylid, rhacophorid or dicroglossid tadpoles. The panels show impacts on (a) survival rate, (b) body length, (c) body mass, and (d) developmental stage (Gosner 1960). The graphs show mean values (based on 6 replicate containers per treatment) with associated standard errors. Mi = microhylid, Rh = rhacophorid; Di = dicroglossid tadpoles.

After 20 days, survival rates of cane toad tadpoles ranged from 56 to 80% per treatment. Surprisingly, survival rates were higher in the treatment with 20 toad tadpoles than with 10 toad tadpoles (z = 2.04, p = 0.042; Fig 1A). Survival rates in the 40 *R*. *marina* and 10 *M*. *ornata* treatments were also higher than in the 10 *R*. *marina* treatment, although the differences were marginally non-significant (z = 1.76, p = 0.08, z = 1.82, p = 0.07; respectively; see Fig 1A). Survival rates of toad tadpoles exposed to 10 *F*. *sakishimensis* or 10 *R*. *owstoni* tadpoles were not significantly different from those recorded in the 10 *R*. *marina* treatment (z = 0.93, p = 0.35, z = 0.19, p = 0.85; respectively; Fig 1A).

Differences in body size as a result of experimental treatment were more clearcut. Treatment affected snout-vent length (F_5, 30_ = 23.90, p < 0.0001), mass (F_5, 30_ = 27.87, p = 0.0001) and developmental stage (F_5, 30_ = 7.37, p < 0.0001) of cane toad tadpoles at 20 days of age (Fig 1B, 1C and 1D). For each of these three descriptors, posthoc Tukey HSD tests show that the effect of adding 10 Microhylid tadpoles was similar to that of adding an additional 10 cane toad tadpoles. However, the addition of 10 Rhacophorid or Dicroglossid tadpoles significantly reduced toad tadpole performance below that seen with 10 toad tadpoles alone, or with 10 Microhylids present. The effect of adding 10 Rhacophorid tadpoles was to reduce toad SVL and mass to a level equivalent to (or lower than) that seen if we added an additional 10 toad tadpoles (i.e., the “20 *R*. *marina*” treatment). Toad developmental rate was even more sensitive, with the addition of 10 Rhacophorid tadpoles reducing toad developmental rates as much as seen after adding an additional 30 toad tadpoles (“40 *R*. *marina*” treatment). Ten Dicroglossid tadpoles reduced toad SVL and mass to a level equivalent to that seen in higher densities of toads (“40 *R*. *marina*” treatment), significantly greater than the effect of an additional 10 toad tadpoles (“20 *R*. *marina*” treatment). The negative impact of 10 Dicroglossid tadpoles on toad developmental rate was equivalent to that induced by 30 toad tadpoles. That is, Dicroglossid and Rhacophorid tadpoles exerted a greater per-capita suppressive effect on toad tadpole body length, mass and developmental rate than did Bufonid or Microhylid tadpoles.

Toad tadpole SVL and mass were highly correlated (p < 0.0001, r^2^ = 0.82). There was a significant treatment effect on tadpole body shape (ANOVA on residual scores from the general linear regression of ln mass on ln snout-vent length: F_5, 30_ = 3.90, *P* = 0.008). Post-hoc Tukey HSD tests showed the addition of Microhylid or Rhacophorid tadpoles had no significant effect on body shape of toad tadpoles. However, toad tadpoles were more slender-bodied if exposed to 10 Dicroglossid tadpoles or to higher densities of toad tadpoles (20, 40 tadpoles), than in other treatments.

## Discussion

At least in the laboratory, cane toad tadpoles exhibit markedly reduced rates of growth and/or development if raised with the tadpoles of Dicroglossid and Rhacophorid species that occur on Ishigaki Island. Microhylid tadpoles also suppress toad viability, but to a lesser extent (only to a similar degree as do conspecific toad tadpoles). That is, toad tadpoles develop most rapidly if they are raised at low densities, without other tadpoles–but increasing the numbers of tadpoles per container (regardless of whether they are toads or native frogs) markedly reduced the rates of growth and development of toad tadpoles. If similar suppressive effects occur in the field, competition within the larval phase may constitute a significant form of biotic resistance to the cane toad’s colonisation of Ishigaki Island.

Our results broadly mirror those of Cabrera-Guzmán et al., who studied interactions between invasive cane toads and eight species of native Australian frogs, using methods very similar to our own [11]. Those authors reported strong interspecific suppression of cane toad larvae (albeit, variable among species), sometimes involving rates of survival as well as growth and development. Larger native tadpoles had more impact than smaller tadpoles, consistent with our own study (in which the smallest species, the Microhylid, had the least effect on toad tadpoles). Generally, larger tadpoles are competitively superior to smaller tadpoles [20–22]. This size effect may result from higher food-consumption rates of larger tadpoles, perhaps combined with behavioural avoidance of larger animals by the smaller toad larvae [11, 23].

As well as absolute size, the impact of native tadpoles on cane toads may be affected by habitat use. The Microhylid tadpoles in our experiments spent most of their time close to the water surface, whereas the Bufonids, Dicroglossids and Rhacophorids were primarily bottom-dwellers. The difference in habitat use may further reduce competition between Bufonid and Microhylid larvae. More generally, we might expect native species to thrive under local conditions (to which they have presumably become adapted over long periods of evolutionary history) whereas an invasive species such as the cane toad has had a much briefer period to adapt to those conditions.

Biotic resistance, although common, is not universal. For example, a study from Florida reported no negative effects of two species of native frogs (a Bufonid and a Hylid) on survival or time to metamorphosis in invasive cane toads [14]. Given the interspecific diversity in impacts evident both in our own study and that of Cabrera-Guzmán et al. [11], it would be premature to draw any general conclusions about geographic effects. Future work on a wide range of species would be of great interest.

If we understand the nature and magnitude of interactions between introduced species and native taxa, we may be able to exploit that understanding to develop novel environmentally-friendly approaches to invader control. Most obviously, we can increase densities of native taxa in waterbodies likely to be used for spawning by invasive anurans [11]. Our results suggest that this approach may be effective in areas of subtropical Japan to which cane toads have been introduced, as well as the mainland Australian sites that have been the focus of most previous study.
